# Supplementary material for: Differential virulence of Trypanosoma brucei rhodesiense isolates does not influence the outcome of treatment with anti-trypanosomal drugs in the mouse model
Source: PLoS One. 2020 Nov 5;15(11):e0229060. doi: 10.1371/journal.pone.0229060 (PMC7643984; doi:10.1371/journal.pone.0229060)
Supplement: S1 Fig — (i): Mean survival times in mice infected with the very-acute clones of Trypanosoma brucei rhodesiense. (ii): Mean survival times in mice infected with the acute clones of Trypanosoma brucei rhodesiense. (iii): Mean survival times in mice infected with the sub-acute clones of Trypanosoma brucei rhodesiense. (iv): Mean survival times in mice infected with the chronic clones of Trypanosoma brucei rhodesiense. (DOCX) [file pone.0229060.s001.docx]

**S1 Fig**

**
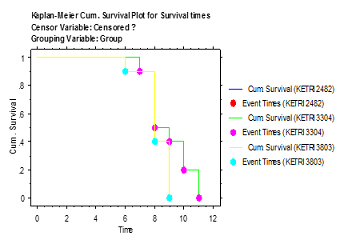
**

S1 Fig (i): Mean **s**urvival times in mice infected with the very-acute clones of *Trypanosoma brucei rhodesiense.*


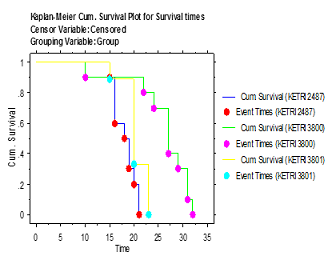


S1 Fig (ii): Mean **s**urvival times in mice infected with the acute clones of *Trypanosoma brucei rhodesiense.*

*
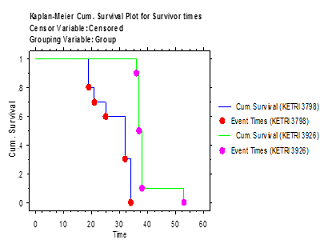
*

S1 Fig (iii): Mean **s**urvival times in mice infected with the sub-acute clones of *Trypanosoma brucei rhodesiense*

*
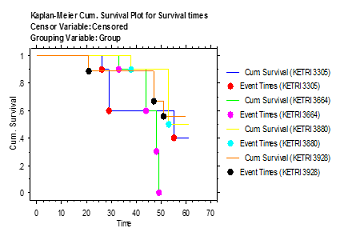
*

S1 Fig (iv) : Mean **s**urvival times in mice infected with the chronic clones of *Trypanosoma brucei rhodesiense*
