# Supplementary material for: Differential virulence of Trypanosoma brucei rhodesiense isolates does not influence the outcome of treatment with anti-trypanosomal drugs in the mouse model
Source: PLoS One. 2020 Nov 5;15(11):e0229060. doi: 10.1371/journal.pone.0229060 (PMC7643984; doi:10.1371/journal.pone.0229060)
Supplement: Attachment — Submitted filename: Response to reviewers comments.docx [file pone.0229060.s005.docx]

| # | **Reviewers comments** | **Response** |
| --- | --- | --- |
|  | Why was the control group (not infected mice) not included in Table 1. Although this group is not mentioned in Materials and Methods, it is mentioned in results in lines 200 and 213. This would add more credence to the results. | We concur with the reviewer’s suggestion and have therefore added the following sentence to the materials and methods section “A control group of 10 non-infected mice was included in the study and monitored for changes in PCV, body weight and survival times” *”margin lines 127-128. We have also included the control group in table 1 of the marked manuscript margin line 263-264”* |
|  | In line 188, please mention the groups that the mice infected with very acute clone were being compared to. | We concur with the reviewer. In the revised manuscript, this statement has been re-phrased to read as follows: *“*When parasitaemia data for *T. b. rhodesiense* clones in each virulence class were grouped together and compared, parasitaemia was significantly (p<0.001) higher in mice infected with very- acute clones*, ”*margin line 251-254of the marked manuscript. |
|  | We noticed you have some minor occurrence(s) of overlapping text with the following previous publication(s), which needs to be addressed: <https://doi.org/10.1371/journal.pntd.0001857> <https://doi.org/10.1017/S0031182017002359> <https://www.afro.who.int/health-topics/trypanosomiasis-african> | We have made changes to the manuscript to address the issue of overlapping texts. Where the text in question needs to be maintained, we have provided appropriate citations as follows:   1) <https://doi.org/10.1371/journal.pntd.0001857>.   We have modified our text to read as follows:  According to Maclean et al (2008), the pathology encountered in the acute HAT infections is characterized by elevated Tumor necrosis factor alpha (TNF-α) while that encountered in the chronic HAT infections is characterized by elevated transforming growth factor (TGF-β) *(Line 66-68)*   2) <https://doi.org/10.1017/S0031182017002359>   In the revised manuscript, the affected section now reads as follows “Each mouse’s survival time was determined on the basis of a ≥ 25% decline in PCV and consistently high parasitaemia levels of 1x10^9^/ml for at least two consecutive days as previously described by Kamidi et al,2018 [26]” (margin lines 141-145 of the marked manuscript).The work by Kamidi et al, 2018 was done in the same laboratory in which the present study was done, hence similar methods were used to determine survival time.  3. <https://www.afro.who.int/health-topics/trypanosomiasis-african>  We have improved our text to read ‘’ The sensitivity of these isolates to suramin and melarsoprol is significant since currently they are the only drugs of choice recommended by WHO (2018) to treat early and late stages of *Tbr* HAT respectively’’. Lines 420-423 of the marked manuscript |
|  | Please amend your list of authors on the manuscript to ensure that each author is linked to an affiliation. Authors’ affiliations should reflect the institution where the work was done (if authors moved subsequently, you can also list the new affiliation stating “current affiliation:….” as necessary) | We acknowledge this observation by the reviewer and has hence forth corrected this in the revised manuscript. *“Margin lines 8,13 and 15 of the marked manuscript”* |
|  | Please upload a new copy of Figure 1 as the detail is not clear. Please follow the link for more information: [http://blogs.PLOS.org/everyone/2011/05/10/how-to-check-your-manuscript-image-quality-in-editorial-manager/](http://blogs.plos.org/everyone/2011/05/10/how-to-check-your-manuscript-image-quality-in-editorial-manager/) | A new copy of this figure has been uploaded |
|  | Please include captions for your Supporting Information files at the end of your manuscript, and update any in-text citations to match accordingly. Please see our Supporting Information guidelines for more information: <http://journals.plos.org/plosone/s/supporting-information>. | We concur with the reviewer. Supporting information captions are now included at the end of the manuscript as suggested. |
|  | Reviewer # 1 |  |
| 1 | Figures 1 to 4 have data that combines observations from different clones grouped together. Because there is no specific information on the 12 clones, all scientific data is lost, and no external reader can see what exactly is going on. All figures should be split into the 4 groups described, and for each group, all individual clone data should be provided. The figure legend should include how many mice were used to obtain data for each clone, and how many repeats were done. Without this information, the paper is just a 'cartoon'. With this data, this reviewer is convinced the paper could have a big impact and could open the door even for collaborative work, as it seems the authors have a very valuable unique collection in their hands. But, for that the scientific data has to be there so it is clear for every clone what is exactly happening. | We appreciate this observation by the reviewer. For each of the figures 1-4, we have now provided data for each of the clones as supporting information as follows: survival (Fig 1(i)-(iv) in S1, Parasitaemia Fig 2 (i)-(iv) in S2, PCV Fig 3 (i)-(iv) in S3 and body weight Fig 4(i)-(iv) in S4 and updated the relevant sections of the text as follows: survival data (margin lines 185-203), parasitaemia (margin lines 232-252), PCV (margin lines 279-287) and body weight (margin lines 297-303) of the revised manuscript.  The number of mice used in each virulence study is now provided in the legend as was suggested by the reviewer (survival, line 203, parasitaemia, line 255, PCV, line 293 and body weight, line 304) |
| 2 | The authors started with 12 stabilates and selected from every stabilate a single clone. In the discussion, they 'walk' backwards and suggest that the individual virulence of each clone, could reflect different levels of virulence of human infections. In order to do that, a crucial experiment is missing. In order to make such conclusion one should take different clones from one and the same, and show that within a stabilate, all clones have the same level of virulence. This is actually very unlikely because it is known that every field stabilate contains a whole collection of different parasites expressing different VSGs and possibly even using different expression sites. Taking one clone per stabilate, and just assuming that this clone represent the behavior of the entire population has no scientific basis at all. Hence, any conclusion based on such assumption is juts scientifically wrong. Please remove that speculation section from the discussion because it undermines the intellectual value of the paper. | We thank the reviewer for this comments. We have addressed the reviewer’s comments in two ways:   1) Expanding on our explanation why we used cloned stabilates instead of field stabilates. In the revised manuscript, the sentence reads “We used *T. b rhodesiense* clones because they represent a homogeneous population of genetically identical trypanosomes thus making subsequent studies based on these clones more reproducible and reliable as previously reported (lines 345-347) 2) We have deleted reference to clinical profiles of HAT as suggested by the reviewer (*margin lines 358-359*). 3) We have also included the statement ’However, we recommend a similar study be carried out using the parent primary isolates (margin lines (449-452) of the revised manuscript. |
|  | Reviewer # 2 |  |
| 1 | It would be helpful to non-specialist readers if the authors included a few sentences remarking on the clinical relevance (or lack thereof) of the mouse model for virulence and drug resistance of T. brucei in humans. For example, is there any reason to expect that isolates that are exceptionally virulent in mice will be also be exceptionally virulent in humans and vice versa? The authors do demonstrate that Tbr isolates previously characterized as sensitive or resistant remain so using the mouse assays described in this paper, but similar controls are not provided for virulence. | We concur with the reviewer’s suggestions and have therefore inserted in the statement “Studies on disease pathogenesis, parasite virulence, drug sensitivity and identification of new potential drug targets and staging biomarkers are commonly carried out in the mouse model based on its cost effectiveness, genetic similarity to humans estimated to be 97.5%, and ethical limitations of carrying out such studies in higher animal models or humans [14,15,16] .” margin lines 80 -84 of the marked manuscript  Direct comparison of parasite virulence data obtained using inbred mice (minimal host variation) and data obtained from clinical cases of HAT is difficult due to host variations in humans. Therefore, since the use of inbred mice is designed to minimize/eliminate the effect of host variations on disease outcome, it is common to infer that parasites found to be virulent in mouse model would also be virulent in humans.  No controls were provided for virulence because the idea for the current study was to compare parasite virulence with drug sensitivity patterns. |
| 2 | In lines 277-278, the authors remark that there is no correlation between virulence and number of passages since isolation and they cite Table 2 as evidence, but I fail to see how the information in Table 2 makes this point. Furthermore, it would appear that the very acute isolates are mostly older than the less acute isolates. Is there evidence of a reduction in the virulence of Tbr in recent years, perhaps because of enhanced genetic drift as the number of Tbr infections decreases? | We appreciate the reviewers comment. We would, however, like to clarify that it was never our intention to state that there is no correlation between virulence and number of passages. Rather, the intended meaning was that since the study isolates had undergone only a minimal number of passages, the observed differences in virulence are likely caused by intrinsic attributes of the trypanosome. In the revised manuscript, the relevant sections reads as follows: “The majority (11/12) of the *Tbr* clones used in this study had undergone a minimal number of passages (1-8) passages since isolation (Table 2). The only isolate that had undergone a significant (64) number of passages (Table 2) did not exhibit notably different virulence characteristics from the other clones in the class, suggesting therefore that the observed differences in isolate virulence is an intrinsic attribute as previously reported [36] (lines 366-369) of the revised manuscript. Table 1 has been modified to show the number of passages of each trypanosome isolate (margin line 263-264). There is no data comparing virulence between older and recent *Tbr* isolates and there will be need to undertake such a study in future. |
| 3 | In lines 236-238, the authors remark that several of the mice treated with diminazene acetate died "due to causes not related to trypanosome infection." Please provide more details, e.g., what was the cause of death? In light of the small number of mice (5) used to assess resistance, it is somewhat concerning that so many of them (14) died from unrelated causes. | We are in agreement with the reviewer and have rephrased the sentence to read as follows:…. died at 47 days post treatment and without parasitologically detectable trypanosome (Table 3), This is presumably due to pneumonia resulting from cold environment caused by a leaking water bottle. *“margin lines 325-328 of the marked manuscript”* |
| 4 | This study suggests that virulence is not a strong predictor of drug resistance for Tbr sampled over this time scale from these particular locations. However, the significance of this result is somewhat weakened by the fact that none of the tested isolates exhibited resistance. Are there data from other locations that suggest that such a correlation sometimes exists? | We appreciate this concern raised by the reviewer. This has now been improved in the revised manuscript and reads as follows” Overall, the fact that the test isolates were all sensitive (at least 80% cure rates) to the drugs suggests there was no relationship between isolates’ virulence and their sensitivity to anti-trypanosomal drugs which is in agreement with previous studies [46,47]. In a study by Sokolova et al, [48]these authors observed that resistance to nifurtimox did not compromise parasite virulence. This is despite previous studies reporting that drug resistant trypanosome have reduced virulence [49,50]. (margin lines 430-435). |
| 5 | In Table 2, Sensitive Isolate KETRI 2537 is shown as resistant to MelB at the lowest drug does (1 mg/kg) despite the fact that all 5 mice are shown as being cured. Is this a data entry error? If not, please explain the result. | We welcome this observation by the reviewer and has as a results corrected our data to read 0/5 implying all the 5 mice were resistant to MelB 1mg/kg (margin line 331-332) of the marked manuscript. |
| line 45: | two subspecies | This is now corrected as suggested by the reviewer *“margin line 47 of the marked manuscript”* |
| line 47 | tsetse flies | This now corrected as suggested by the reviewer *“margin lines 49 of the marked manuscript”* |
| line 53 | two stages, namely the early (hemolymphatic) and the late (meningo-encephalitic) stage. | This now corrected as suggested by the reviewer *“margin line 55 of the marked manuscript”* |
| line 59 | as well as anemia | Corrected as suggested by the reviewer *“margin line 61 of the marked manuscript”* |
| line 72 | In their study, Pyana and colleagues [13] | Corrected as suggested by the reviewer *“margin lines 74-75 of the marked manuscript”* |
| line 86 | 6- to 8-week old | Corrected as suggested *“margin line 93 of the marked manuscript”* |
| line 114 | What do you mean by "ip"? | This is an abbreviation of intraperitoneal as described on *margin line 112 of the marked manuscript* |
| line 130 | The classification of trypanosome virulence was based | Corrected as suggested *“margin line 138 of the marked manuscript”* |
| line 141 | determined for Melarsoprol | Corrected as suggested *“margin line 151 of the marked manuscript”* |
| lines 149 and 151 | 10 ml | Corrected as suggested *“margin lines 161, 162 and 165 of the marked manuscript”* |
| line 160 | if there are | Deleted the word exist and rephrased the sentence to read ….. if there are significant differences….. “*margin line 170 of the marked manuscript”* |
| line 170 | exhibited variation | Corrected as suggested *“margin line 182 of the marked manuscript”* |
| line 185 | were not statistically significant | Corrected as suggested by the reviewer *“margin line 231 of the marked manuscript”* |
| line 258 | [27] does not appear to be the correct reference for this statement about Tbr genetic variation | We wish to clarify that this reference [27] is in the material and method section on determination of survival and has no reference to genetic variation *(margin lines 145 -146 of the marked manuscript)*.Statement on genetic variation is in the discussion section and is supported by reference [30] *margin line 346-347*. |
| line 259 | the existence of variation in virulence among | Corrected as suggested *“margin line 347 of the marked manuscript”* |
| line 261 | Uganda | Corrected as suggested *“margin line 351 of the marked manuscript”* |
| line 310 | Our results showed a decline in body weight in the early days of infection (7 - 14 dpi) followed by a recovery except in mice infected with very acute strains. | Corrected as suggested *“margin lines 410-411of the marked manuscript”* |
| line 312 | confirms a previous observation | Corrected as suggested *“margin line 412 of marked manuscript”* |
| line 318 | I'm not sure what you mean by "with days post infection"? | We concur with the reviewer that this statement is ambiguous and is now deleted *“margin line 418 of the marked manuscript”* |
| line 341 | there is variation in virulence | Corrected as suggested *“margin line 445 of the marked manuscript”* |
| line 348 | Other studies are needed to confirm | This has been rephrased to read as follows “Since our study was based on laboratory cloned *T. b. rhodesiense* stabilates, however, future studies should utilize the parent primary isolates”.  *“ margin line 449-451 of the marked manuscript”* |
