# Supplementary material for: Differential virulence of Trypanosoma brucei rhodesiense isolates does not influence the outcome of treatment with anti-trypanosomal drugs in the mouse model
Source: PLoS One. 2020 Nov 5;15(11):e0229060. doi: 10.1371/journal.pone.0229060 (PMC7643984; doi:10.1371/journal.pone.0229060)
Supplement: Attachment — Submitted filename: Response to reviewer # 2 comments.docx [file pone.0229060.s006.docx]

**Reviewer #2:**

The authors have successfully addressed all of the major concerns raised in my original review and the resulting study makes an interesting contribution to our understanding virulence and drug resistance in African trypanosomiasis. However, there are still some minor grammatical errors and one questionable claim in the revised manuscript, as listed below. Note that the line numbers given here refer to the unmarked revised text in the file sent for review, not the marked revised text that appears in the second half of the file.

.

| line 91: The authors remark that the mouse has a "genetic similarity to humans estimated to be 97.5%",but I don't know any way of defining genetic similarity that would give such a high number. According to the website <https://www.genome.gov/10001345/importance-of-mouse-genome>, the average similarity between the shared protein-coding genes is around 85%. Please delete or modify this claim. | We thank the reviewer for this observation and has modified this claim as suggested. It now reads ‘’……. genetic similarity to humans estimated to be 85 %,……..’’  *Margin line 95 of the marked manuscript* |
| --- | --- |
| line 45: A cure rate of at least 80% was achieved for all test isolates | This has now been rephrased to read as suggested. *Margin lines 45-46 of the marked manuscript* |
| line 48: evidence of variation in ... confirms that this variation is | We have corrected this as suggested. *Margin line 50 of the marked manuscript.* |
| line 73: in different foci differ in both their | Corrected as suggested*. Margin line 74 –marked manuscript* |
| line 87: this variation | Corrected as suggested. *Margin line 88-marked manuscript.* |
| line 143: once a week using a | Corrected as suggested. *Margin lines 144-145- marked manuscript* |
| line 146: The classification of trypanosome virulence | Corrected as suggested. Margin line 147-marked manuscript |
| line 165: were prepared | Corrected. *Margin line 166* |
| line 179: body weight changes | Corrected. *Margin line 180* |
| line 181: The general linear model in SAS was ... between the means of the four virulence classes. | Corrected as suggested. *Margin lines 182-183* |
| line 190: exhibited variation in | Corrected. Margin line 191 |
| line 196: Mice infected with the acute clones had MST ranging from 18.1 +- 0.7 (KETRI 2487) to 26 +- 2.0 for... (You don't need to keep repeating the phrase mean +- SEM for each set of numbers.) | In agreement with the reviewer and has deleted the repeated phrase mean +- SEM. *Margin lines 197-198* |
| line 207: The overall MST was 8.7 +- for the very acute clones, 21.6 +- 1.0 for the acute clones, | Corrected as suggested. *Margin lines 207-209 –marked manuscript.* |
| line 227: are shown in Table and Figure 2(v). | Corrected *.margin line 229-marked manuscript* |
| line 231: clone specific variation was observed | Corrected as suggested. *Margin line 233* |
| line 257: The pre-infection PCV data for all infected | Corrected. *Margin 259* |
| line 258: The PCV of the infected mice declined significantly following infection when compared | Corrected as suggested. Margin line 260-261-*marked manuscript.* |
| line 308: at least 80% cure rates for all drug dose regimens | Corrected. *Margin line 310-marked manuscript* |
| line 309: a few cases of relapse | Corrected. *Margin line 311-marked manuscript.* |
| line 314: without detectable parasites. | Corrected. *Margin 316- marked manuscript* |
| line 343: a previous report of variation in virulence | Corrected. *Margin line 345-marked manuscript* |
| line 356: differences in isolate virulence are an intrinsic attribute | Corrected. margin 358- marked manuscript |
| line 357: Parasitemia progression differed significantly among Tbr isolates assigned to different virulence classes on the basis of survival time | Corrected as suggested. *Margin line 359-360-marked manuscript* |
| line 363: acute infections result from | Corrected Margin line 366- *marked manuscript.* |
| line 371: they reveal that the majority | Corrected. *Margin line 374-marked manuscript.* |
| line 391: on the causes of body weight changes in animals infected with trypanosomes, especially after | Corrected. *Margin line 393-394-marked manuscript* |
| line 413: due to the mutation or absence of a drug uptake gene | Corrected. *Margin line 416* |
| line 419: In summary, this study has found that there is variation in | Corrected. *Margin line 422-marked manuscript.* |
